# Polysaccharide Multilayer Films in Sensors for Detecting Prostate Tumor Cells Based on Hyaluronan-CD44 Interactions

**DOI:** 10.3390/cells9061563

**Published:** 2020-06-26

**Authors:** João Batista Maia Rocha Neto, Andrey Coatrini Soares, Rogério Aparecido Bataglioli, Olívia Carr, Carlos Alberto Rodrigues Costa, Osvaldo N. Oliveira, Marisa Masumi Beppu, Hernandes F. Carvalho

**Affiliations:** 1 School of Chemical Engineering, University of Campinas, 13083-852 Campinas, Brazil; jbmrneto@gmail.com (J.B.M.R.N.); rogerbataglioli@gmail.com (R.A.B.); beppu@unicamp.br (M.M.B.); 2 National Laboratory of Nanotechnology for Agribusiness (LNNA), Embrapa Instrumentation, 13560-970 São Carlos, Brazil; andreycoatrini@gmail.com; 3 São Carlos Institute of Physics, University of São Paulo,13566-590 São Carlos, Brazil; oliviacarr@gmail.com (O.C.); chu@ifsc.usp.br (O.N.O.J.); 4 Brazilian Nanotechnology National Laboratory (LNNano), Brazilian Center for Research in Energy and Materials (CNPEM), Campinas, 13083-970 São Paulo, Brazil; carlos.costa@lnnano.cnpem.br; 5 Institute of Biology, University of Campinas, 13083-970 Campinas, Brazil; 6 National Institute of Photonics Applied to Cell Biology (INFABIC), 13083-859 Campinas, Brazil

**Keywords:** hyaluronan, cancer, CD44 receptor, sensing, layer-by-layer films, information visualization

## Abstract

The increasing need for point-of-care diagnosis has sparked the development of label-free sensing platforms, some of which are based on impedance measurements with biological cells. Here, interdigitated electrodes were functionalized with layer-by-layer (LbL) films of hyaluronan (HA) and chitosan (CHI) to detect prostatic tumor cells (PC3 line). The deposition of LbL films was confirmed with atomic force microscopy and polarization-modulated infrared reflection absorption spectroscopy (PM-IRRAS), which featured the vibrational modes of the HA top layer capable of interacting specifically with glycoprotein CD44 receptors overexpressed in tumor cells. Though the CHI/HA LbL films cannot be considered as a traditional biosensor due to their limited selectivity, it was possible to distinguish prostate tumor cells in the range from 50 to 600 cells/µL in in vitro experiments with impedance spectroscopy. This was achieved by treating the impedance data with information visualization methods, which confirmed the distinguishing ability of the films by observing the absence of false positives in a series of control experiments. The CD44–HA interactions may, therefore, be exploited in clinical analyses and point-of-care diagnostics for cancer, particularly if computational methods are used to process the data.

## 1. Introduction

Hyaluronic acid (HA) (also referred to as hyaluronan or hyaluronate) is a linear glycosaminoglycan comprising repeating disaccharide units of d-glucuronic acid (1-β-3) *N*-acetyl-d-glucosamine (1-β-4) with a molecular weight from 10^4^ to 10^8^ Da, depending on its origin [1]. It is found throughout the body but most concentrated in the vitreous of the eye [2], in the synovial fluid, and in the extracellular matrix of cartilages [3]. HA is also produced by microorganisms such as *Streptococcus equi* and *Streptococcus zooepidermicus* [4]. Physiologically, HA is responsible for a structural role in cartilages [5], as it is also relevant for protein homeostasis in the extracellular space [6], and lubrication of joints and tissues due to its rheological properties [5]. Cell processes involving HA include proliferation [7], locomotion [8], recognition, and differentiation [9]. The biocompatible properties of HA have been explored for clinical applications, especially in building blocks for the design of advanced materials. Several of these applications rely on the interaction of HA with cell surface receptors such as CD44, which are a family of receptor proteins in the plasma membrane of leukocytes and other cells [10]. Führmann and co-workers described the role of CD44-HA interactions for the survival and differentiation of pluripotent stem cells derived from oligodendrocyte progenitor cells on injectable hydrogels of peptide-modified hyaluronan and methylcellulose [11]. When injected in an injured rat spinal cord, grafted cells in the hydrogel mostly differentiated to a glial phenotype with adequate levels of survival and integration and attenuated teratoma formation. Hence, HA-containing hydrogels may be suitable for treating lesions in the central nervous system with minimal invasion [11]. Swiston and co-workers created hybrid patches that can be attached to the surface of lymphocytes through CD44-HA interactions, which could be used for drug delivery and imaging [12,13].

The overexpression of the CD44H isoform, which contains a specific binding domain to HA in different carcinomas, gliomas, and non-Hodgkin’s lymphomas [10], indicates that CD44-HA interactions can be exploited to capture circulating tumor cells (CTC) for diagnostic purposes, though some of the CD44 isoforms do not bind HA [10]. Indeed, HA-functionalized surfaces have been used to capture prostatic cancer cells with biospecific HA-CD44 interactions [14,15]. Detection of CTC is considered a label-free strategy for prostate cancer diagnosis since the number of CTC is a reliable marker to predict tumor response and survival [16,17], even at early stages. CTC detection has been carried out using different approaches. For example, antibody-based methods capture CTC from heterogeneous samples by targeting over-expressed molecules on cell membranes such as epithelial cell adhesion molecules (epCAM) [18] and prostate-specific membrane antigen (PSMA). Another strategy focuses on the identification of pre-selected RNA markers using reverse transcriptase/polymerase chain reactions (RT/PCR) [19]. These strategies based on CTC detection are complementary to those involving the determination of prostate cancer biomarkers such as prostate-specific antigen (PSA) [20,21,22] and prostate cancer antigen 3 (PCA3) [23,24,25].

In this paper, we report on the detection of prostate cancer (PC3 line) cells using layer-by-layer (LbL) films [26] containing HA to take advantage of biospecific HA-CD44 interactions. In the LbL films, HA layers are alternated with chitosan (CHI) layers that are known to be suitable matrices for sensors and biosensors [22]. The LbL method is especially useful for sensing because it enables the fine-tuning of film properties according to the materials and process conditions for surface functionalization [27]. In the experiments described herein, the LbL film growth was monitored using polarization-modulated infrared reflection absorption spectroscopy (PM-IRRAS) and atomic force microscopy (AFM). Detection was performed using impedance spectroscopy measurements, and the data were treated with an information visualization method [25,28].

## 2. Materials and Methods

### 2.1. Materials

Hyaluronic acid (HA, ~1500–1800 kDa) extracted from *Streptococcus equi*, low molecular weight chitosan (CHI) (deacetylation degree > 85%), polyethyleneimine (PEI, MW ~7.5 KDa), fetal bovine serum (FBS), Dulbecco’s phosphate-buffered saline (DPBS), and phosphate-buffered saline (PBS) were purchased from Sigma Aldrich, Saint Louis, USA. HA and PEI were dissolved in ultrapure water (18.2 MΩ cm of resistivity at 25 °C) and stirred for 24 h to prepare 0.1% (*w*/*v*) solutions. CHI was dissolved in acetic acid (100 mM) and stirred for 24 h to obtain a 0.1% (*w*/*v*) solution. HCl (1 M) and NaOH (1 M) were added to the polyelectrolyte solutions to adjust their pH to 3.0. The PC3 tumor cell line was obtained from the American Type Culture Collection (ATCC, Manassas, USA). Streptomycin/penicillin (S/P, 5000 U.I./mL) and HAM-F12K cell culture media were purchased from Lonza (Basel, Switzerland).

### 2.2. Electrode Preparation

Gold interdigitated electrodes were fabricated onto BK7 substrates (6 × 6 cm) at the Brazilian Nanotechnology National Laboratory (LNNano) following the methods previously described [25]. The electrodes had 50 pairs of 10 μm wide digits spaced 10 μm away from each other. They were sequentially washed in an ultrasonic bath with isopropanol and ethanol, both followed by one rinsing step in deionized water for 5 min. The electrodes were then exposed to O_2_ plasma (Harrick Plasma Cleaner, PDC-32G) at 100 mTorr, 720 V DC, 25 mA DC, 18 W, for 10 min to remove organic materials. This also favors stronger electrostatic interactions with weak polyelectrolytes by increasing the number of hydroxyl groups on the glass surface [29].

### 2.3. Electrode Modification

Before depositing the natural polymers, the electrodes were immersed in the PEI solution (0.1% *w*/*v*, pH 4, 100 mM NaCl) for 10 min to create a monolayer with a high charge surface density, which increases the homogeneity of subsequent polyelectrolyte layers. Next, the multilayers were assembled by alternating immersions of the electrodes (10 min each) in HA and CHI solutions, respectively, with three rinsing steps with ultrapure water (2 min, 1 min, and 1 min, respectively) between each immersion in the polyelectrolyte solutions. Films containing 3.5 bilayers were produced with HA on the outermost layer and subsequently dried at room temperature. Electrodes were selected based on their similarity in terms of electrical impedance spectra for the electrode modification step, which was performed automatically, thereby assuring reproducible performance on the impedance measurements.

### 2.4. Film Characterization

The electrode modification was confirmed with atomic force microscopy (AFM), Kelvin probe force microscopy (KPFM), and polarization-modulated infrared reflection absorption spectroscopy (PM-IRRAS). Topography and surface potential images were acquired simultaneously using a Park NX-10 Atomic Force Microscope (South Korea) in the tapping mode setup, with an electric AC signal set at 17 kHz to the metal-coated cantilever. The measurements were performed under air by single-pass scanning at room temperature (25 °C) and humidity of approximately 5%. Image and data analysis were carried out using Gwyddion open-source software. CHI/HA functionalization was monitored by the presence of polyelectrolyte functional groups at the electrode using PM-IRRAS acquired with a KSV spectrophotometer (model PMI 550, KSV Instruments, Helsinki, Finland), with an incident angle of 81° and spectral resolution of 8 cm^−1^. The PM-IRRAS signal was taken from the reflectivity of the components *s* and *p* through Equation (1),

(1) $$\frac{\Delta R}{R}=\frac{R_{p}-R_{s}}{R_{p}+R_{s}}$$

where *R_p_* is the reflectivity of the parallel component, and *R_s_* is the reflectivity of the component perpendicular to the plane of incidence of the incoming IR light.

### 2.5. Detection of Tumor Cells

Functionalized interdigitated electrodes were exposed to different concentrations of tumor cells (500, 1000, 3000, 10,000, and 15,000 cells mL^−1^) for 1 h at 37 °C and then washed with phosphate-buffered saline (PBS) solution to remove the non-adhered cells. Subsequently, the prostatic tumor cells (PC3 line) adhered to the electrode surface were detected using electrical impedance spectroscopy with a Solartron model SI 1260 A (Solartron Analytical, Hampshire, UK) in the range between 1 and 10^6^ Hz. Electrical measurements were performed in triplicate and collected to plot the capacitance spectra. Electrical impedance data were also acquired for electrodes without any modification, i.e., without the layer-by-layer film, and with functionalized electrodes just exposed to PBS solution and others containing ascorbic acid, glucose, and fetal bovine serum (FBS) as control experiments. PC3 cells were maintained at 37 °C with 5% CO_2_ in HAM-F12K media containing 10% FBS and 1% streptomycin/penicillin. The sensing data were treated by applying information visualization methods, which provided optimization and evaluation of the sensing performance. The free software PEx-Sensors [30] was used to run the projection technique interactive document mapping (IDMAP) [31,32,33] to evaluate selectivity. Cell adhesion tests with the CHI/HA films assembled onto glass substrates were performed as described in [15] with a 1 h incubation step and a subsequent rinsing step with PBS solution to remove non-adhered cells. The adhered cells were fixed in 2% (*w*/*v*) paraformaldehyde (15 min) and washed with DPBS, and then their actin filaments were stained with TRITC-phalloidin (1:500). Micrographs were acquired using the Axio Observer.Z1 Zeiss inverted confocal L510 microscope (Carl Zeiss AG, Germany) and the objectives Plan-Apochromat 10× and 63×/1.4 oil.

## 3. Results and Discussion

### Physicochemical Characterization

**Figure 1:**
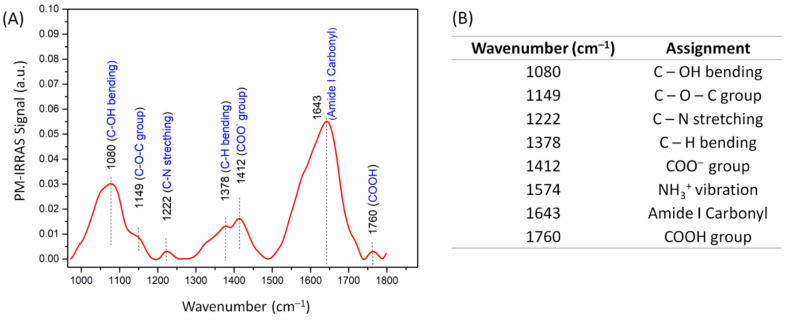
(**A**) Polarization-modulated infrared reflection absorption spectroscopy (PM-IRRAS) spectrum of gold substrates functionalized with hyaluronic acid and chitosan. The spectrum for the uncoated substrate was used as the baseline. (**B**) Assignment of the main bands in the PM-IRRAS spectrum.

The capture of PC3 tumor cells is driven by specific interactions between their CD44 receptors and HA molecules [14], and therefore, it is relevant to confirm the presence of carboxyl groups from HA in the LbL film. Electrode modification was confirmed by the PM-IRRAS spectrum in Figure 1A, with the band assignment given in Figure 1B. The bands at 1412 cm^−1^ and 1760 cm^−1^ are assigned to carbonyl stretching in the carboxylate (COO^−^) and carboxylic acid (COOH) groups from HA, respectively [34]. Hence, the carboxylate (deprotonated) groups in HA chains may interact with amino groups from CHI, establishing charge reversal that promotes film assembly [35]. The broad absorption band at 1080 cm^−1^ is due to vibrational modes associated with C–OH bending in HA molecules [34]. Both polysaccharides exhibit a broad absorption between 1800 and 1600 cm^−1^, which contains several characteristic absorptions from their chains. The band at 1643 cm^−1^ is assigned to the asymmetric stretching of amide I carbonyl groups and to the carbonyl stretching from carboxylate groups [34,36]. These C–N stretching and C–OH bending bands confirm the presence of HA in the film [36,37]. The strong absorption at 1650 cm^−1^ might contain contributions from −NH_2_ scissoring vibrations of amine groups from CHI [38]. The spectrum also displays bands at 1574 cm^−1^ and at 1149 cm^−1^, assigned to symmetric −NH_3_^+^ vibration [38] and to the ether bonds in HA and CHI [34,36], respectively.

**Figure 2:**
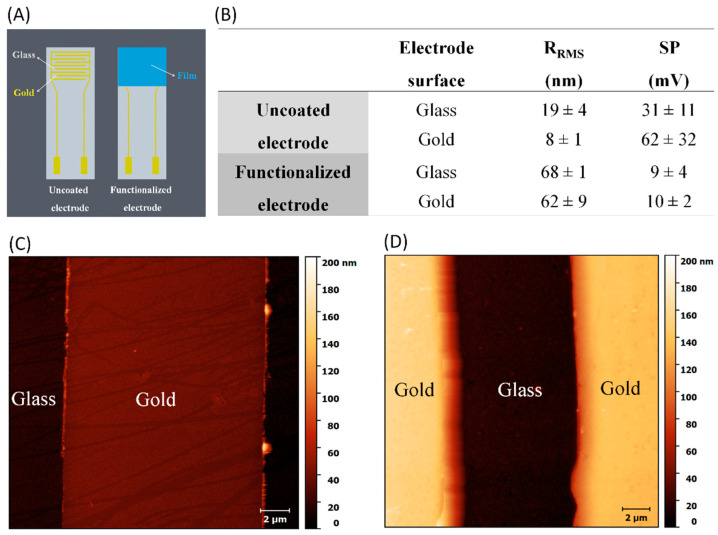
(**A**) Schematic representation of the electrode functionalization. (**B**) Average measurements of root mean square roughness (R_RMS_) and surface potential (SP) for electrodes before and after layer-by-layer (LbL) functionalization. Atomic force microscopy (AFM) images for interdigitated electrodes (**C**) before and (**D**) after chitosan (CHI)/hyaluronan (HA) film deposition.

The AFM images in Figure 2C,D and the KPFM images in Figure S1 (Supplementary Materials) indicate that CHI/HA multilayers increased the average root mean square roughness (R_RMS_) and surface potential, respectively. These values are shown in Figure 2B, where one notes that R_RMS_ increased from 19 and 8 nm for uncoated glass and gold surfaces to 68 and 62 nm with LbL functionalization, respectively. The AFM image in Figure 2B shows the typical morphology of weak polyelectrolyte films with a buildup of isolated islets [14,39,40]. As expected from the literature, the LbL deposition of non-conducting natural polymers induced a decrease in surface potential (SP) on the interdigitated electrode surface [41,42]. After electrode modification, no significant differences in terms of surface potential and roughness were observed for the functionalized electrode surface. This reinforces the successful multilayer assembly inferred from the PM-IRRAS data.

**Figure 3:**
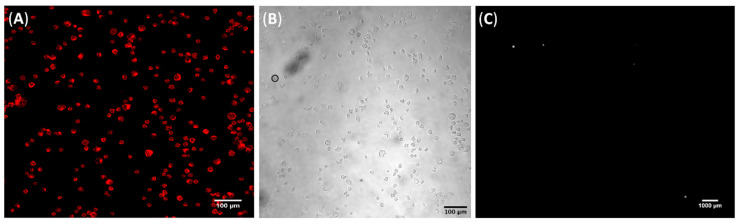
Representative micrographs of (**A**) TRITC-phalloidin stained PC3 cells attached to a CHI/HA film deposited on a glass substrate and (**B**) its respective bright field image. Scale bars are 100 µm for both. (**C**) Representative dark field micrograph of PC3 cells attached to an uncoated glass substrate. Scale bar is 1000 µm.

Understanding cell–substrate interactions is vital to developing biomaterials for diagnostics, and this may be achieved by monitoring cell adhesion transduced into electrical signals. This approach may help to determine the mechanisms underlying the interaction between the CD44 receptor and HA. Cells adhered to electrodes may be considered as circuit elements, where the impedance changes in a frequency-dependent manner as cells attach to the electrodes [43]. In this study, the electrodes were coated with 3.5 multilayers of CHI/HA using the LbL technique under optimized film assembly conditions (pH 3.0 and 100 mM of NaCl) [15]. A careful electrode selection process based on similar electrical impedance measurements preceded film deposition to avoid noise and interference due to manufacturing defects. The functionalized electrode had HA as the top layer, and the ionic strength and pH of the polyelectrolyte solutions were controlled to modulate HA availability and, consequently, tumor cell capture. In addition to using gold interdigitated electrodes, we deposited LbL films on glass substrates to monitor whether cell adhesion would also occur on a different substrate as a proof of concept. Figure 3 shows the micrographs of PC3 cells adhered to CHI/HA films on glass after 1 h of contact, reinforcing the role of HA as the direct mediator of the cell adhesion mechanism.

**Figure 4:**
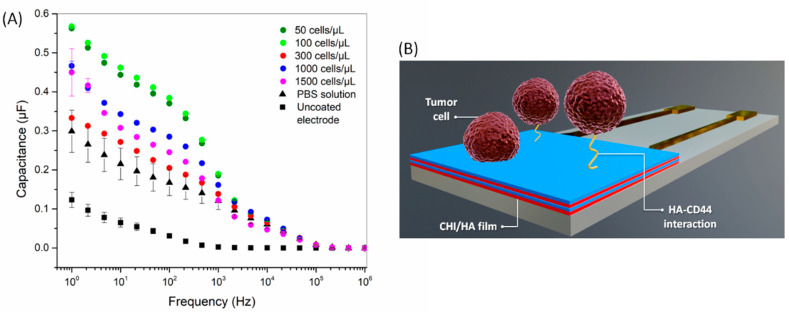
(**A**) Capacitance spectra for CHI/HA functionalized electrodes after exposure to different concentrations of tumor cells (50–1500 cells/µL). The spectrum of an uncoated electrode is also shown. (**B**) Schematic representation of tumor cell adhesion mediated by CD44-HA interaction in CHI/HA functionalized electrodes.

The detection of tumor cells adhered to the electrode surface after 1 h of contact was evaluated with electrical impedance spectroscopy after dropping 10 µL of PBS. Before the electrical measurements, the functionalized electrodes were gently rinsed with PBS to remove non-adhered cells and the culture media. The electrical response depends on a combined effect of the double-layer capacitance, the membrane resistance, and the impedance of the medium [43,44]. At low frequencies, the adhered cell behavior is driven by electric dipoles owing to the migration of oppositely charged ions in the cell, while at high frequencies, the electrical signal is dominated by cell spreading and electrode geometry [43]. The capacitance spectra in Figure 4 show prominent changes at low to intermediate frequencies (1–10 kHz); the figure shows the specific interaction mediates the sensor response between CD44 receptors of tumor cells and HA on the electrode. This induces changes in the electrical double layer at the interface between the film and PBS solution [44,45]. Indeed, we recently reported the role of hyaluronan and CD44 receptor availability on the modulation of cell-substrate adhesion properties of CHI/HA films [15], which are consistent with the capacitance results.

The difference in capacitance induced by exposure to different concentrations of cells is apparent at a fixed frequency (10 Hz, for example), which is evidence of the detection capability of CHI/HA functionalized electrodes. As the impedance spectra may be sensitive even to small changes on the electrode (or sensor) surface, additional strategies are necessary to visualize the data when many samples are analyzed. A refined analysis was performed here using information visualization techniques, which reduce the dimensionality of the sensing data and enable the evaluation of selectivity from similarity/dissimilarity in the data. The capacitance spectra of tumor cells were analyzed with the IDMAP technique, which projects each measured spectrum in a point on the 2D map, with similarity/dissimilarity defined by Equation (2):

(2) $$S_{IDMAP}=\frac{\delta \left(x_{i},x_{j}\right)-\delta_{\min}}{\delta_{\max}-\delta_{\min}}-d\left(y_{i},y_{j}\right)$$

where $\delta_{\max}$/$\delta_{\min}$ are maximum/minimum Euclidean distances between the data instances, and $\delta \left(x_{i},x_{j}\right)$ and $d\left(y_{i},y_{j}\right)$ are Euclidean distances in the original and lower-dimensional space, respectively.

**Figure 5:**
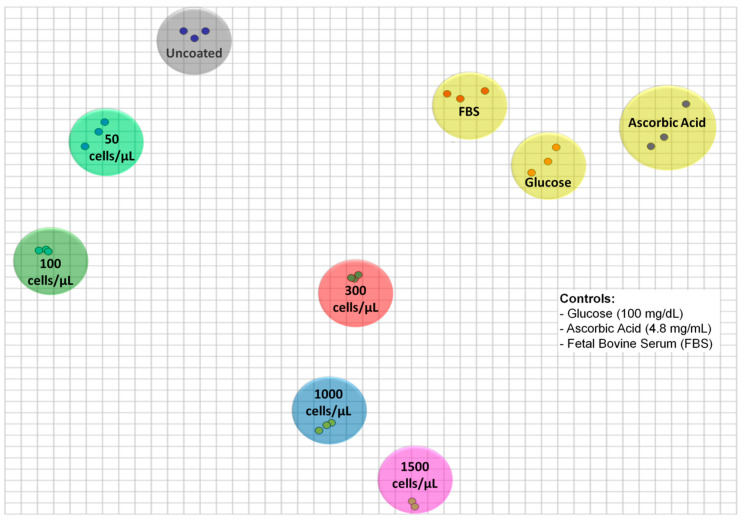
Two-dimensional interactive document mapping (IDMAP) plot for the capacitance spectra for uncoated electrodes and 3.5 CHI/HA-multilayer functionalized electrodes exposed to different tumor cell concentrations (50–1500 cells/µL) and non-specific analytes (glucose (100 mg/dL), ascorbic acid (4.8 mg/mL), and fetal bovine serum (FBS)).

In order to generate the 2D IDMAP plot of Figure 5, the Euclidean distances between each pair of spectra in Figure 4 were input into Equation (2) to determine S_IDMAP_, whose values were eventually placed on the 2D plot. The IDMAP plot shows a clear distinction of different concentrations of tumor cells, which were located on distinct clusters. It is significant that the points related to increased tumor cell concentrations are placed from top to bottom. Additionally, saturation of the CHI/HA sensor response occurs between 1000 and 1500 cells/µL, as is normal in sensors (or biosensors) on which the number of available sites for interaction vanishes with increasing concentration. The selectivity of the CHI/HA functionalized electrodes was evaluated in three control experiments, with exposure to samples containing ascorbic acid, glucose, and fetal bovine serum (FBS) (Figure S2). The data points from these control experiments are close together and far from the points of the tumor cells in Figure 5. The absence of false positives in the latter results indicates that the sensitivity of the sensor arises mainly from CD44-HA interactions.

The distinguishing ability of the sensor can be estimated using the silhouette coefficient (S) calculated according to Equation (3) [30],

(3) $$S=\frac{1}{n}\sum_{i=1}^{n}\frac{\left(b_{i}-a_{i}\right)}{\max\left(b_{i},a_{i}\right)}$$

where *n* is the number of samples, *a_i_* is the average distance calculated between the i_th_ spectrum projection of the tumor cells and the remaining projections for the capacitance spectra of tumor cells, and *b_i_* is the minimum distance of its projection and projections of other clusters with different tumor cell concentrations. This coefficient varies between −1 and 1, in which values near 1 indicate a high distinction ability between the clusters, and values near 0 and −1 indicate that the data do not assist in distinguishing the samples. The CHI/HA sensors presented a high analytical performance with a silhouette coefficient of 0.791, which is comparable to that of biosensors to detect new prostate (CaP) biomarkers [25].

As promising as the results in Figure 5 may be, one cannot be sure that the selectivity of CHI/HA films is irrefutable. For example, the top layer made of HA allows for adsorption of a further CHI layer, and this could affect the impedance (or capacitance spectra). We have tested this hypothesis by measuring the spectra for LbL films containing 1.5 bilayers (i.e., HA/CHI/HA), 3.5 bilayers, and 5.5 bilayers with no tumor cells and noted slight differences among them (Figure S3). However, when the data of all samples and control experiments were projected on an IDMAP plot (Figure S4), the distinction of the tumor cells at different concentrations was still clear. Hence, it seems that one may be able to distinguish non-specific adsorption on the CHI/HA LbL films from the changes induced by HA-CD44 interactions.

## 4. Conclusions

The ability of hyaluronan (HA) to capture tumor cells that overexpress CD44 receptors has been exploited here for detection purposes. This was made possible by measuring the impedance spectra of LbL films of HA and chitosan (CHI) deposited on interdigitated electrodes. We refer to these films as forming sensors (and not biosensors) because their selectivity is limited. Indeed, we observed that the capacitance spectra of the CHI/HA LbL films depend on the number of bilayers and can be slightly affected by exposure to analytes other than the tumor cells. However, the distinguishing ability of the CHI/HA sensors was clear in in vitro experiments for prostatic tumor cells at concentrations from 50 to 600 cells/µL. Such ability was demonstrated with the multidimensional projection technique IDMAP and a silhouette coefficient of 0.791. It could be attributed to the specific interactions between HA and CD44, as the functional groups of HA on the top layer of LbL films could be identified in PM-IRRAS measurements.

Our results serve as a proof of concept that CHI/HA LbL films have the potential for the selective capture of tumor cells in a fast, simple, and label-free screening procedure, in which the HA chain acts as the direct mediator of the cell adhesion mechanism. The limitation mentioned here associated with the limited selectivity can be addressed with computational methods, such as the multidimensional projection techniques employed here or machine learning methods for classification [33,45] for even more demanding tasks in which non-specific adsorption is likely to be a problem. Furthermore, one may utilize a sensing array rather than just one sensing unit, e.g., with different numbers of bilayers in the LbL films, as in the concept of electronic tongues that can also be used in biosensing [46,47]. With such strategies, it will be possible to extend the use of HA-containing films to capture other types of tumor cells when there is overexpression of CD44 receptors.

## Supplementary Materials

The following are available online at https://www.mdpi.com/2073-4409/9/6/1563/s1, Figure S1: Kelvin probe force microscopy (KPFM) images for interdigitated electrodes before and after deposition of 3.5 bilayers of CHI/HA bilayers; Figure S2: Capacitance spectra for electrodes functionalized with 3.5 CHI/HA bilayers exposed to non-specific analytes, ascorbic acid, and fetal bovine serum; Figure S3: Capacitance spectra for an uncoated interdigitated electrode and electrodes functionalized with 1.5, 3.5, and 5.5 CHI/HA bilayers; Figure S4: 2D DMAP plot for the capacitance spectra for uncoated electrodes and 3.5 CHI/HA-multilayer functionalized electrodes exposed to different tumor cell concentrations and non-specific analytes, ascorbic acid, and fetal bovine serum.
