# Supplementary material for: Polysaccharide Multilayer Films in Sensors for Detecting Prostate Tumor Cells Based on Hyaluronan-CD44 Interactions
Source: Cells. 2020 Jun 26;9(6):1563. doi: 10.3390/cells9061563 (PMC7349506; doi:10.3390/cells9061563)
Supplement: Supplementary file 1 [file cells-09-01563-s001.pdf]

Polysaccharide multilayer films in sensors for detecting prostate tumor cells based on hyaluronan-CD44 interactions

J. B. M. Rocha Neto, A. C. Soares, R. A. Bataglioli, O. Carr, C. A. R. Costa,

O. N. Oliveira Jr[3], M. M. Beppu[1], H. F. Carvalho*

*e-mail: hern@unicamp.br

Supporting information

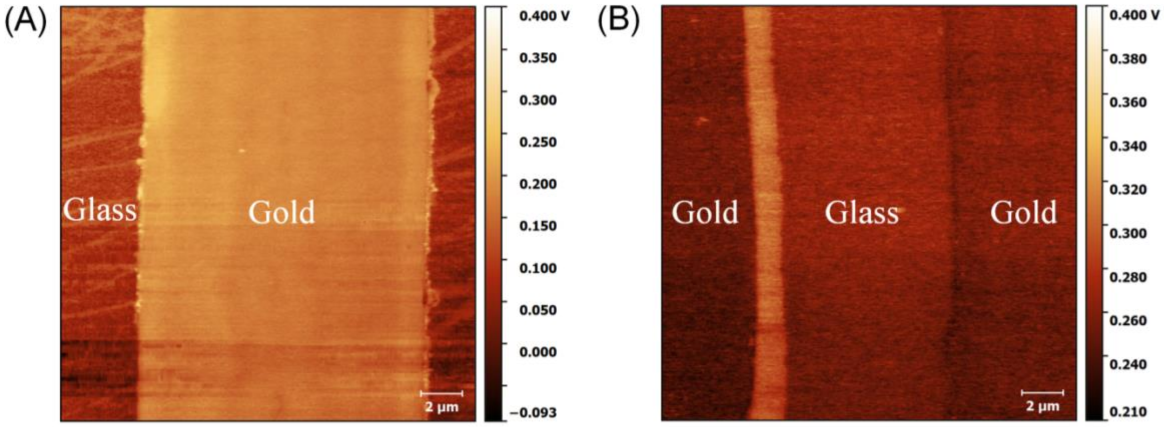

Figure S1. KPFM images for interdigitated electrodes **(A)** before and **(B)** after deposition of 3.5 bilayers of CHI/HA bilayers. The surface potentials for gold and gold coated with the LbL film are (62 ± 32) mV and (10 ± 2) mV, respectively. The roughness values were (31 ± 11) nm and (9 ± 4) nm for glass and glass coated with the LbL film, respectively.

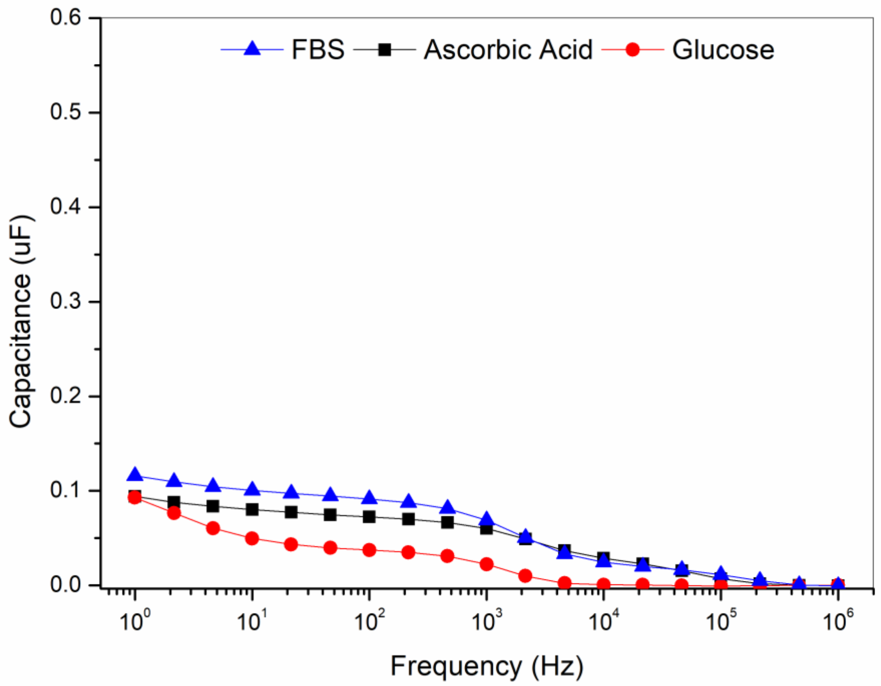

Figure S2. Capacitance spectra for electrodes functionalized with 3.5 CHI/HA bilayers exposed to non-specific analytes (glucose (100 mg/dL), ascorbic acid (4.8 mg/mL) and fetal bovine serum (FBS).

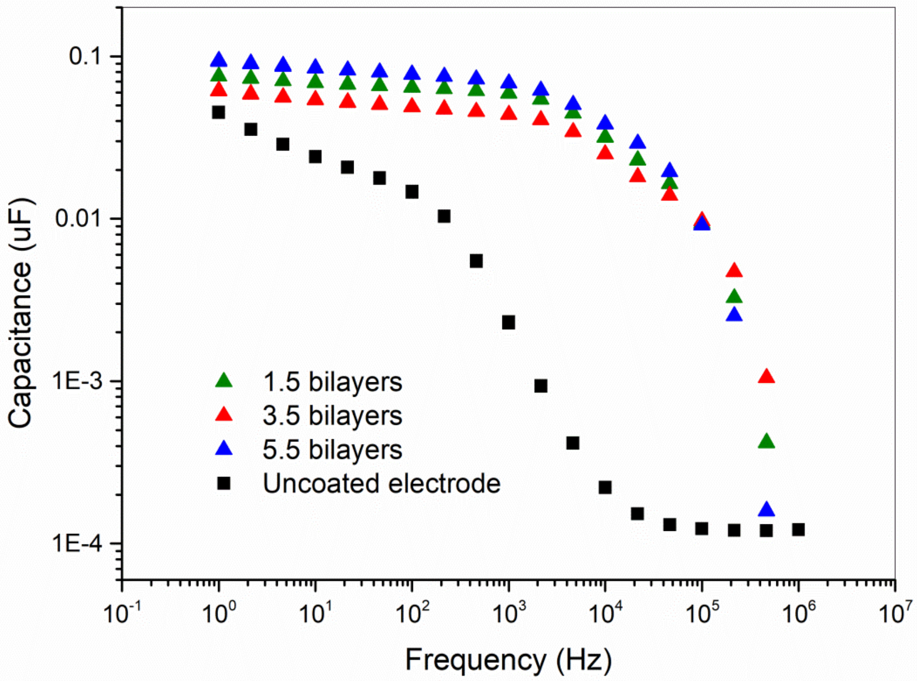

Figure S3. Capacitance spectra for an uncoated interdigitated electrode and electrodes functionalized with 1.5, 3.5 and 5.5 CHI/HA bilayers.

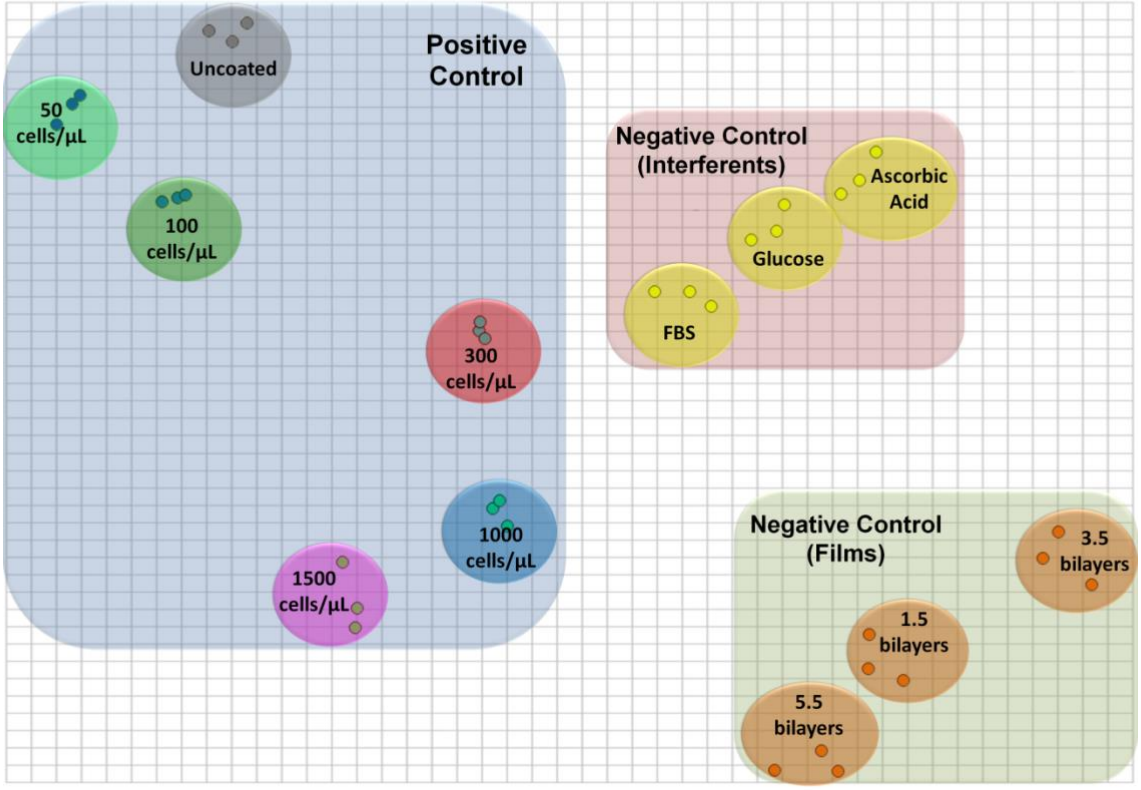

Figure S4. 2D DMAP plot for the capacitance spectra for uncoated electrodes and 3.5 CHI/HA multilayers functionalized electrodes exposed to different tumor cell concentrations (50-1500 cells/µL) and non-specific analytes (glucose (100 mg/dL), ascorbic acid (4.8 mg/mL) and fetal bovine serum (FBS)). Here, a second control experiment was performed in PBS solution with electrodes covered with different number of HA/CHI bilayers (1.5, 3.5 and 5.5).
